# Daratumumab-based treatment of monoclonal gammopathy–associated angioedema due to acquired C1-inhibitor deficiency

**DOI:** 10.1016/j.jacig.2024.100322

**Published:** 2024-08-05

**Authors:** Remy S. Petersen, Lauré M. Fijen, Laurens E. Franssen, Josephine M.I. Vos, Danny M. Cohn

**Affiliations:** a Department of Vascular Medicine, Amsterdam Cardiovascular Sciences, Amsterdam University Medical Center, University of Amsterdam, Amsterdam, The Netherlands; b Department of Hematology, Jeroen Bosch Hospital, Den Bosch, The Netherlands; c Department of Immunohematology Diagnostics, Sanquin Diagnostic Services, Amsterdam, The Netherlands; d Department of Hematology, Cancer Center Amsterdam and Amsterdam Institute for Infection and Immunity, Amsterdam University Medical Center, University of Amsterdam, Amsterdam, The Netherlands

**Keywords:** Acquired C1-inhibitor deficiency, angioedema, monoclonal gammopathy, MGUS, daratumumab, bradykinin

## Abstract

Daratumumab-based treatment could control severe, treatment-refractory, life-threatening angioedema due to acquired C1-inhibitor deficiency associated with monoclonal gammopathy.

Angioedema due to acquired C1-inhibitor deficiency (AAE-C1INH) is a very rare but serious condition that has an estimated prevalence of 1 in 500,000 individuals and is characterized by unpredictable, debilitating, and potentially life-threatening bouts of swelling.^1^ AAE-C1INH typically arises in persons older than 40 years and is thought to result either from increased clearance of C1-inhibitor (C1INH) by autoantibodies, which are identified in 47% to 67% of patients,^2^ or from increased consumption of C1INH leading to activation of the classical complement pathway by anti-idiotype antibodies. A deficiency of C1INH leads to insufficient inhibition of the kallikrein-kinin system and subsequent excessive production of bradykinin, inducing increased vascular permeability and formation of angioedema. Anti-CD20 treatment with rituximab (with or without coadministration of cyclophosphamide) is reported to effectively restore C1INH functionality in most patients, even when no associated conditions are identified.^3^ Additionally, off-label use of therapies developed for hereditary angioedema have been reported to be effective in reducing attack frequency.^1^^,^^2^ Long-term control of disease can also be achieved through treatment of associated conditions that have been implicated in the development of AAE-C1INH, including autoimmune and B-cell proliferative disorders such as monoclonal gammopathy.^1^ Monoclonal gammopathy is a common premalignant condition. Found in 1% to 2% percent of adults, it is typically asymptomatic and often diagnosed through the incidental finding of a serum monoclonal protein (M-protein) after diagnostic exclusion of lymphoproliferative disorders. Treatment of the underlying clonal disease is indicated only in cases of monoclonal gammopathy of clinical significance (MGCS), when the small clone (serum M-protein level < 3 g/dL and <10% monoclonal plasma cell infiltration of bone marrow) leads to symptoms via a pathologic paraprotein or other mechanisms.^4^

Here, we report a case of severe, refractory, monoclonal gammopathy–associated AAE-C1INH that was successfully treated with daratumumab-based clone-directed therapy.

An 84-year-old male was referred to our outpatient clinic because of uncontrolled AAE-C1INH with recurrent, severe attacks of angioedema, including laryngeal and abdominal attacks. The first episodes occurred when the patient was 80 years old, and the diagnosis was established 1 year thereafter following a life-threatening episode of laryngeal angioedema that was refractory to corticosteroids and adrenaline and necessitated resuscitation and a tracheostomy. The diagnosis was based on decreased C1INH activity (0.52 U/mL [reference range 0.63-1.82 U/mL] measured 5 days after plasma-derived C1INH treatment), age at onset of angioedema, and a negative family history. Comprehensive diagnostic evaluation revealed an IgGλ monoclonal gammopathy of 3.4 g/L without signs of multiple myeloma. There were no detectable C1INH-specific antibodies present, as determined by ELISA. The angioedema attacks responded well to on-demand treatment with icatibant, a selective antagonist to the bradykinin B2 receptor. Nevertheless, the high attack burden required the use of prophylactic treatment. As illustrated in Fig 1, several prophylactic and curative treatment strategies were initiated, with limited effectiveness. Prophylaxis with danazol (200 mg orally once daily) reduced the attack frequency; however, severe psychological side effects required discontinuation of this treatment. Tranexamic acid (1000 mg orally 3 times daily) was not effective. At first presentation in our tertiary referral hospital, the patient had an estimated attack rate of 2 per month and a C1INH activity of 0.12 U/mL [range 0.63-1.82 U/mL]. Treatment with 4 weekly cycles of rituximab (intravenously at a dose of 375 mg/m^2^) was initiated, without improvement of C1INH activity or attack frequency. Six months later, a regimen of 4 monthly rituximab doses (administered intravenously at a dose of 375 mg/m^2^) with daily cyclophosphamide for 12 weeks (administered orally at a dose of 150 mg) was initiated, again without clinical improvement. Throughout these treatment periods, the patient required escalating doses of prophylactic intravenous plasma-derived C1INH concentrate. Disease control was achieved only after administration of 4000 U of C1INH concentrate per week (administered intravenously in 3 doses).

**Fig 1:**
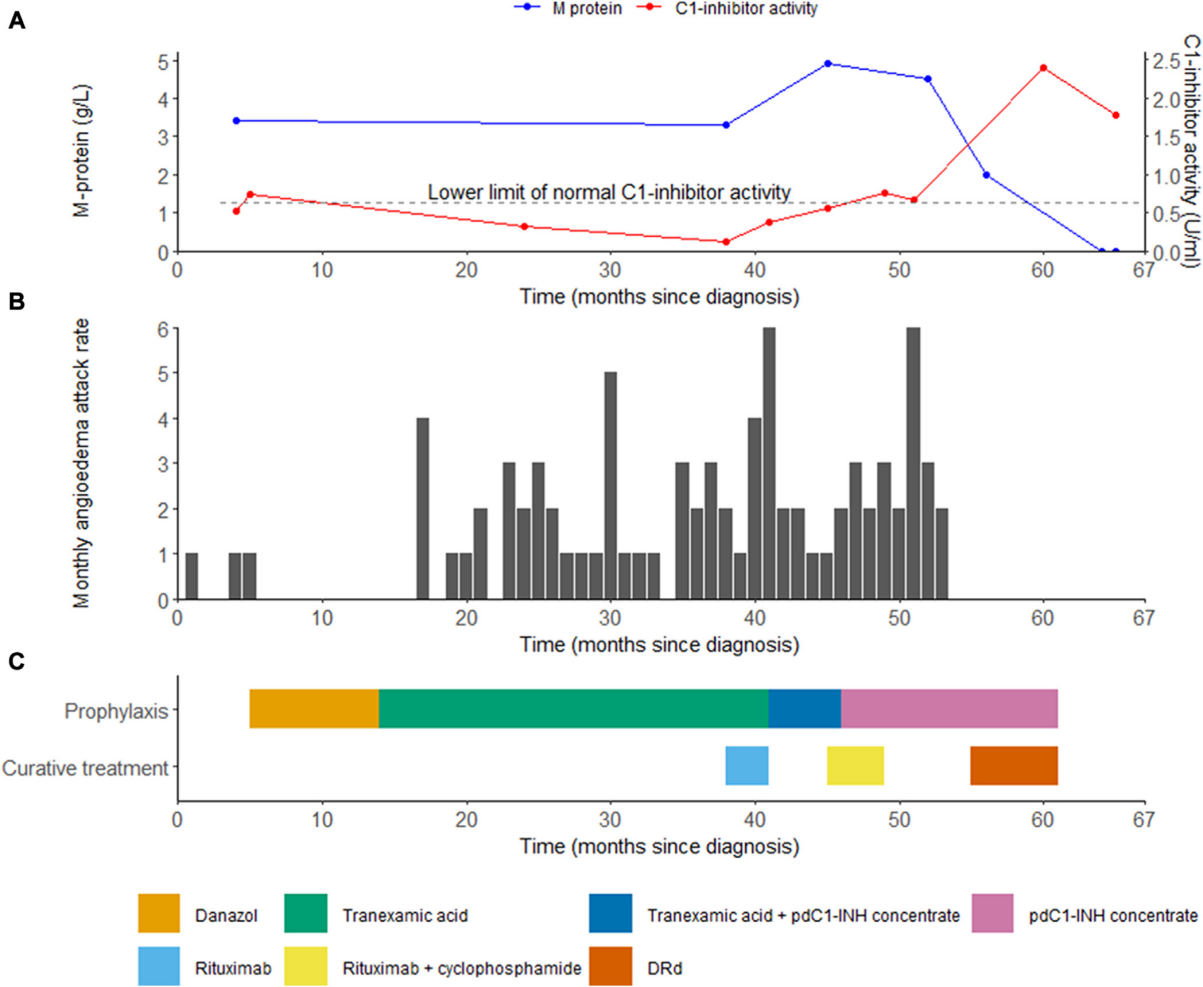
Response to treatment following diagnosis of acquired C1-inhibitor deficiency. **A,** Trends of M-protein (y-axis [*left*]) and functional C1-inhibitor (y-axis [*right*]) levels. **B,** Number of angioedema attacks per month. **C,** Prophylaxis and curative treatment administered during the observation period. *pdC1-INH*, Plasma-derived C1-inhibitor.

Because of this requirement and the significant impact of the attacks on the patient’s quality of life, treatment of the underlying MGCS was considered. Repeated diagnostics did not show signs of progression to malignancy (M-protein level < 3 g/dL, <10% monoclonal plasma cell infiltration in bone marrow analysis, and no end-organ damage). The patient was treated with 6 cycles (4 weeks each) of daratumumab-lenalidomide-dexamethasone (DRd), as illustrated in Fig 2, aimed at eradicating the plasma cell clone. DRd is a first-line treatment regimen for multiple myeloma that combines an anti-CD38 mAb (daratumumab, flat-dose of 1800 mg administered subcutaneously once per week for cycles 1 and 2, then every other week for subsequent cycles) with a targeted immunomodulatory drug (lenalidomide, 10 mg administered orally [dose adjusted for renal function] daily on days 1-21 of every cycle) and a potent corticosteroid (dexamethasone, 20 mg administered orally once per week). This regimen was selected on the basis of its high efficacy with good tolerability in multiple myeloma.^5^ A time-limited treatment (6 cycles of 4 weeks each) as opposed to maintenance therapy was preferred because of the small clone size.

**Fig 2:**
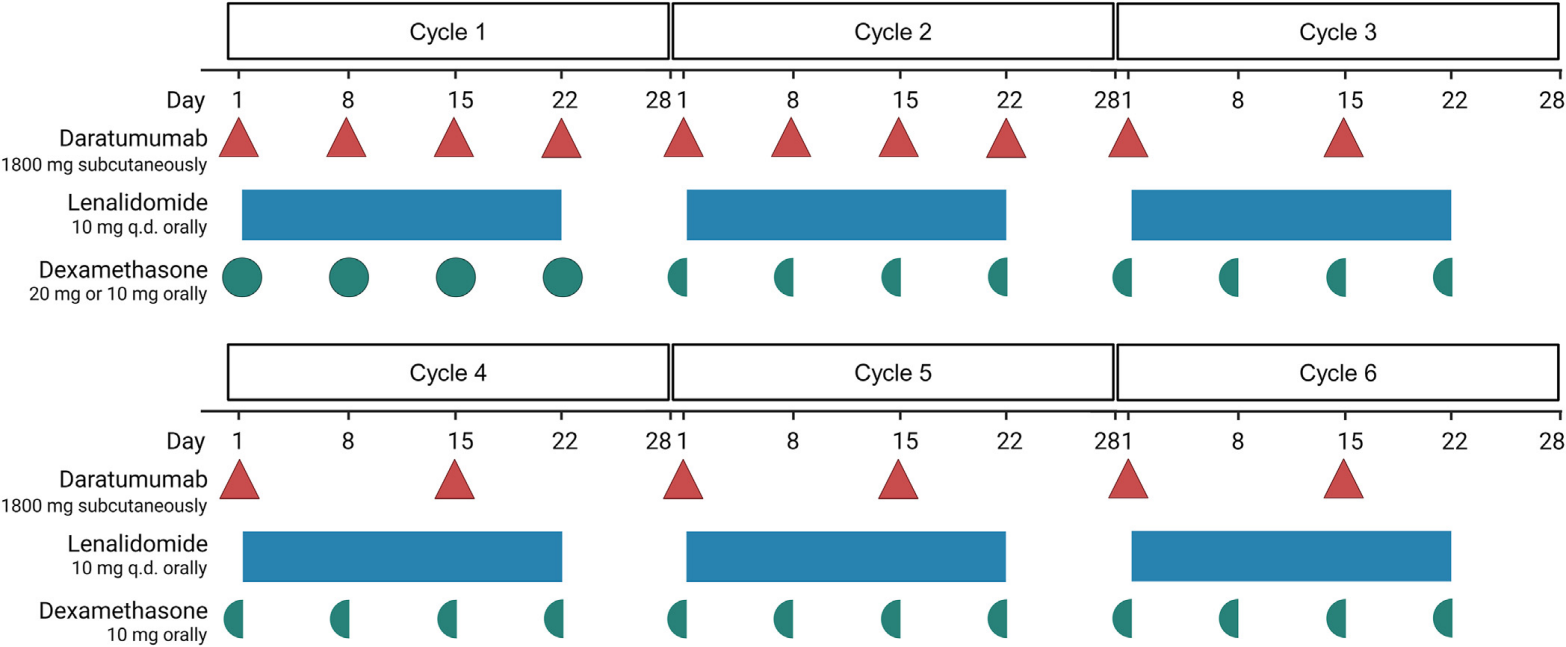
Dosing schedule of DRd cycles 1 through 6. Daratumumab (1800 mg subcutaneously) was administered once per week for cycles 1 and 2 and once every 2 weeks thereafter. Lenalidomide (10 mg orally) was administered daily for the first 3 weeks of every cycle. Dexamethasone was administered orally once every week at 20 mg (cycle 1) and 10 mg (cycles 2-6).

Because of fatigue, insomnia, and emotional lability, the dexamethasone dose was decreased to 10 mg weekly after the first cycle. Thereafter, the patient successfully completed the subsequent 5 cycles with manageable side effects. Following the first cycle, a partial response (≥50% reduction in serum M-protein level) was evident, followed by a very good partial response (≥90% reduction in serum M-protein level) after the second cycle. By the sixth and final cycle, a complete response was achieved, resulting in the eradication of the plasma cell clone with no measurable M-protein. This clinical response was further underscored by the normalization of C1INH activity, and after discontinuing prophylaxis with plasma-derived C1INH concentrate, the patient remained free from attacks during 12 months of follow-up.

This case illustrates the impact of refractory, debilitating, and potentially life-threatening AAE-C1INH as a rare manifestation of monoclonal gammopathy and highlights the potential of daratumumab-based fixed-duration treatment to achieve clinical remission. The positive effect of plasma cell–targeted treatment in our patient supports the involvement of monoclonal gammopathy in the pathophysiology of AAE-C1INH, even in the absence of C1INH-specific antibodies. However, the possibility that this regimen was effective owing to eradication of nonmalignant plasma cells that produced (undetectable) antibodies related to the AAE-C1INH or owing to other immunomodulatory functions of daratumumab, lenalidomide, and/or dexamethasone cannot be excluded.^6^

To our knowledge, this is the first case of AAE-C1INH treated with a daratumumab-based regimen. Some experience exists with daratumumab-based treatment in other forms of MGCS (such as monoclonal gammopathy with renal significance^7^; polyneuropathy, organomegaly, endocrinopathy, monoclonal gammopathy, and skin changes [POEMS] syndrome^8^; and amyloid light-chain amyloidosis).^9^ DRd is a first-line targeted treatment for multiple myeloma; it has a relatively favorable safety profile, although infectious complications and cytopenias may occur. In monoclonal gammopathy–associated AAE-C1INH, daratumumab-based treatment may be considered, specifically, in severe, refractory cases.

## Disclosure statement

Disclosure of potential conflict of interest: R. S. Petersen has received speaking fees from Pharvaris. L. M. Fijen has received a conference travel grant from 10.13039/100013669Ionis Pharmaceuticals and has acted as a consultant for Pharvaris in the past. L. E. Franssen has received hospitality fees from Servier, Celgene, and AbbVie and speaker fees from AbbVie. J. M. I. Vos has received the following as institutional honoraria: research support from Beigene and AbbVie/Genmab; advisory board/consultancy fees from Sanofi and Janssen; and speaker fees from BMS, Sanofi, and Amgen. D. M. Cohn has received speaking fees from CSL Behring, Ionis Pharmaceuticals, Pharvaris, and Takeda; consultancy fees from BioCryst, CSL Behring, Ionis Pharmaceuticals, KalVista, Pharming, Pharvaris, and Takeda; and research support from Ionis Pharmaceuticals, KalVista, Pharvaris, and Takeda.

## References

[1] Cicardi M., Aberer W., Banerji A., Bas M., Bernstein J.A., Bork K. (2014). Classification, diagnosis, and approach to treatment for angioedema: consensus report from the Hereditary Angioedema International Working Group. Allergy.

[2] Baeza M.L., González-Quevedo T., Caballero T., Guilarte M., Lleonart R., Varela S. (2022). Angioedema due to acquired deficiency of C1-inhibitor: a cohort study in Spain and a comparison with other series. J Allergy Clin Immunol Pract.

[3] Levi M., Cohn D., Zeerleder S., Dziadzio M., Longhurst H. (2019). Long-term effects upon rituximab treatment of acquired angioedema due to C1-inhibitor deficiency. Allergy.

[4] Fermand J.P., Bridoux F., Dispenzieri A., Jaccard A., Kyle R.A., Leung N. (2018). Monoclonal gammopathy of clinical significance: a novel concept with therapeutic implications. Blood.

[5] Dimopoulos M.A., Oriol A., Nahi H., San-Miguel J., Bahlis N.J., Usmani S.Z. (2023). Overall survival with daratumumab, lenalidomide, and dexamethasone in previously treated multiple myeloma (POLLUX): a randomized, open-label, phase III trial. J Clin Oncol.

[6] Krejcik J., Casneuf T., Nijhof I.S., Verbist B., Bald J., Plesner T. (2016). Daratumumab depletes CD38+ immune regulatory cells, promotes T-cell expansion, and skews T-cell repertoire in multiple myeloma. Blood.

[7] Zand L., Rajkumar S.V., Leung N., Sethi S., El Ters M., Fervenza F.C. (2021). Safety and efficacy of daratumumab in patients with proliferative GN with monoclonal immunoglobulin deposits. J Am Soc Nephrol.

[8] Gavriatopoulou M., Ntanasis-Stathopoulos I., Fotiou D., Migkou M., Eleutherakis-Papaiakovou E., Kanellias N. (2020). Upfront daratumumab with lenalidomide and dexamethasone for POEMS syndrome. Hemasphere.

[9] Kastritis E., Palladini G., Minnema M.C., Wechalekar A.D., Jaccard A., Lee H.C. (2021). Daratumumab-based treatment for immunoglobulin light-chain amyloidosis. N Engl J Med.

